# Epac: A Promising Therapeutic Target for Vascular Diseases: A Review

**DOI:** 10.3389/fphar.2022.929152

**Published:** 2022-07-14

**Authors:** Yunfeng Pan, Jia Liu, Jiahui Ren, Yun Luo, Xiaobo Sun

**Affiliations:** ^1^ Key Laboratory of Bioactive Substances and Resources Utilization of Chinese Herbal Medicine, Ministry of Education, Institute of Medicinal Plant Development, Chinese Academy of Medical Sciences and Peking Union Medical College, Beijing, China; ^2^ Beijing Key Laboratory of Innovative Drug Discovery of Traditional Chinese Medicine (Natural Medicine) and Translational Medicine, Beijing, China; ^3^ Key Laboratory of Efficacy Evaluation of Chinese Medicine Against Glycolipid Metabolic Disorders, State Administration of Traditional Chinese Medicine, Beijing, China; ^4^ Guizhou University of Traditional Chinese Medicine, Guiyang, China

**Keywords:** Epac, cAMP, vascular disease, therapeutic target, activator and inhibitor

## Abstract

Vascular diseases affect the circulatory system and comprise most human diseases. They cause severe symptoms and affect the quality of life of patients. Recently, since their identification, exchange proteins directly activated by cAMP (Epac) have attracted increasing scientific interest, because of their role in cyclic adenosine monophosphate (cAMP) signaling, a well-known signal transduction pathway. The role of Epac in cardiovascular disease and cancer is extensively studied, whereas their role in kidney disease has not been comprehensively explored yet. In this study, we aimed to review recent studies on the regulatory effects of Epac on various vascular diseases, such as cardiovascular disease, cerebrovascular disease, and cancer. Accumulating evidence has shown that both Epac1 and Epac2 play important roles in vascular diseases under both physiological and pathological conditions. Additionally, there has been an increasing focus on Epac pharmacological modulators. Therefore, we speculated that Epac could serve as a novel therapeutic target for the treatment of vascular diseases.

## Introduction

cAMP is a key secondary messenger that regulates a wide range of biological processes, including cell proliferation (Meroni et al., 2019), hormone release (Yu and Jin, 2010), immunological function (Arumugham and Baldari, 2017), inflammation (Kong X. et al., 2019), and neurological disorders (Bhat et al., 2020). cAMP can induce a wide range of biological effects by activating protein kinase A (PKA), cyclic nucleotide-gated ion channels (CNG) (Kopperud et al., 2003), exchange proteins directly activated by cAMP (Epac) (de Rooij et al., 1998; Kawasaki et al., 1998), phosphodiesterase (PDE) (Gross-Langenhoff et al., 2006), and popeye domain containing (POPDC) proteins (Brand et al., 2014). cAMP/PKA signaling has long been known to play a role in various diseases. Since the discovery of Epac by two independent laboratories in 1998, cAMP/Epac signaling has attracted much attention from researchers. The effector of Epac, Rap, belongs to the Ras superfamily of small G-proteins (de Rooij et al., 1998; Kawasaki et al., 1998). Epac1 and Epac2 (Epac2A, Epac2B, and Epac2C) are the two main subtypes of Epac, encoded by RAPGEF3 and RAPGEF4, respectively (Lee, 2021). Epac1 comprises an auto-inhibitory N-terminal regulatory region and a catalytic region, with multiple domains at the C-terminus. Except for the additional N-terminal cyclic nucleotide-binding domain (CNB), the domains present in Epac1 are identical to those in Epac2 (Kumar et al., 2018; Wehbe et al., 2020). The Ras-exchange motif (REM) domain stabilizes the cell division cycle 25 homology domains (Cdc25-HD), which mediates GDP/GTP exchange activity (Kumar et al., 2018; Wehbe et al., 2020). With increasing levels of intracellular cAMP, the CNB motif binds to cAMP, causing conformational changes in Epac and attenuating CDC25 inhibition. Therefore, downstream Epac effectors, such as Rap1 and Rap2 are activated and involved in GDP/GTP exchange (Tomilin and Pochynyuk, 2019).

Despite their varying levels of expression, Epac1 and Epac2 are found in most tissues. Epac1 is highly expressed in the uterus, ovary, central nervous system, adipose tissue, kidneys, and blood vessels. Epac2A protein is abundant in the brain, pancreas, and pituitary glands. Epac2B and Epac2C proteins are abundant in the adrenal glands and liver, respectively (Hoivik et al., 2013).

Cardiovascular diseases are the leading cause of global mortality (Roth et al., 2020) and have a significant negative impact on human health. Other diseases with vasculopathy, such as cerebrovascular disease (Chu et al., 2021a), renal vascular disease (Halliday and Bax, 2018), and diabetic retinopathy (Sabanayagam et al., 2019), also pose a huge burden on patients and society. Since its discovery in 1998, Epac has attracted considerable interest in scientific research. Compared to therapies targeting the β-adrenergic receptor (β-AR) and adenylyl cyclase (AC), Epac function regulation is expected to ensure a more specific regulation of specific cAMP-mediated signals (Fujita et al., 2017). In this review, we focus on the therapeutic effects of Epac in vascular diseases.

This review focuses on the recent findings regarding the therapeutic effects of Epac in vascular diseases. Most of the references we have included were published during 2016–2021 since there are more innovative achievements during this period. This review focuses on therapeutics for vascular diseases that target the Epac primarily, whereas other studies only concentrating on the specific mechanism or the structure of Epac without relevant disease were excluded. I searched the PubMed database using the keywords “atherosclerosis,” “heart failure,” “arrhythmia,” “ischemia-reperfusion injury,” “stroke,” “Alzheimer’s disease,” “Parkinson’s disease,” “breast cancer,” “glioma,” “lung cancer,” “melanoma,” “pancreatic cancer,” “prostate cancer,” “ischemic kidney injury,” “renal tumor,” “diabetic retinopathy,” “diabetic nephropathy,” and “activator” and “inhibitor” in combination with ‘Epac’. In total, 144 articles were found to be suitable for the topic of this review.

## Cardiovascular Disease

Epac is a key player in the cardio vasculature. Several studies have demonstrated the roles of Epac signaling in the development of cardiovascular diseases (Cai et al., 2016; Jin et al., 2017a; Jin et al., 2018). This review provides an overview of four types of cardiovascular diseases: atherosclerosis, heart failure, arrhythmia, and ischemia-reperfusion injury.

### Atherosclerosis

Atherosclerosis is a complex pathological process that leads to various diseases, such as arrhythmia, angina, and myocardial infarction. Atherosclerosis is characterized by endothelial dysfunction, the proliferation and migration of arterial smooth muscle cells (SMCs), and the formation of foam cells. Inappropriate vascular SMC activation contributes substantially to the development of atherosclerosis (Fuster et al., 2010; Marx et al., 2011). Epac1 is abundantly expressed in the heart and several studies have suggested that it could be used as a therapeutic target for atherosclerosis (Kato et al., 2015; Wang et al., 2016; Robichaux et al., 2020a). A study by Yuko Kato et al. shows that high Epac1 expression levels enhance SMC migration under the stimulation of platelet-derived growth factor-BB, which in turn promotes the development of atherosclerosis. Moreover, Wang et al. showed that the SMC proliferation and PI3K/Akt pathway are suppressed in Epac1^−/−^mice. Moreover, the Epac inhibitor, ESI-09, can reduce neointima formation *in vivo*. These findings validate Epac inhibition as an effective treatment for vascular proliferative diseases (Wang et al., 2016). A recent study showed that Epac1 could promote foam cell formation and atherosclerosis development by upregulating oxidized low-density lipoprotein receptor 1 (LOX1) via stimulation of protein kinase C (PKC), which is an important step of atherosclerosis development. Epac1 downregulation can reduce macrophages and foam cells in atherosclerosis areas (Robichaux et al., 2020b). These findings suggest that inhibition of Epac1 may be a potential strategy for the treatment of atherosclerosis.

### Arrhythmia

Although many organic heart diseases, especially heart failure (HF) and myocardial infarction, can cause arrhythmia, they can also occur on their own. The mechanism of arrhythmia is complex, resulting in the lack of a permanent cure. Therefore, novel therapeutic strategies are urgently required. Fibrotic remodeling plays an important role in atrial fibrillation (Grandoch et al., 2010). Sirirat Surinkaew et al. showed that Epac1-inhibition strategies reduced the left atrial (LA)-fibroblast (FB) collagen secretion (Surinkaew et al., 2019). Moreover, Rajesh Prajapati et al. demonstrated that the sympathetic activation-induced sarcoplasmic reticulum (SR) Ca^2+^ leak was reduced in cardiac myocytes of Epac1^−/−^ mice, and a selective Epac1 inhibitor, CE3F4, can prevent atrial and ventricular arrhythmias in mice (Prajapati et al., 2019a). Zhang MX et al. reported that Epac1 can increase susceptibility to atrial fibrillation by alternating L-type calcium channels (LTCC) in an isoproterenol-induced heart failure (HF) mouse model, which is common in cardiac arrhythmia with an increasingly high risk of stroke and death. Epac1-induced LTCC opening can prolong the action potential (AP) to induce AF, indicating that activating Epac1 can promote AF (Zhang et al., 2019). Moreover, phosphodiesterases2 (PDE2) overexpression protected mice from ventricular arrhythmia induced by ISO by inhibiting the Epac-mediated increases of cellular triggers (Wagner et al., 2021). Therefore, inhibiting the Epac1 signal may be a useful and secure treatment for arrhythmia. Impaired Epac2/Rap1 signaling can cause life-threatening arrhythmia via reactive oxygen species (ROS)-dependent activation of late sodium currents (Yang et al., 2017). Additionally, a map of the genetic changes of the cAMP-signaling cascade in human atria indicated that the Epac2 expression is increased in AF (Garnier et al., 2021). Therefore, besides Epac1, Epac2 can also affect cardiac function in AF. To summarize, the Epac family plays several roles in arrhythmia therapy. Inhibiting Epac1 could be a powerful therapeutic therapy for arrhythmia, and further research is needed to explore the specific role of Epac2 in AF.

### Heart Failure

**FIGURE 1:**
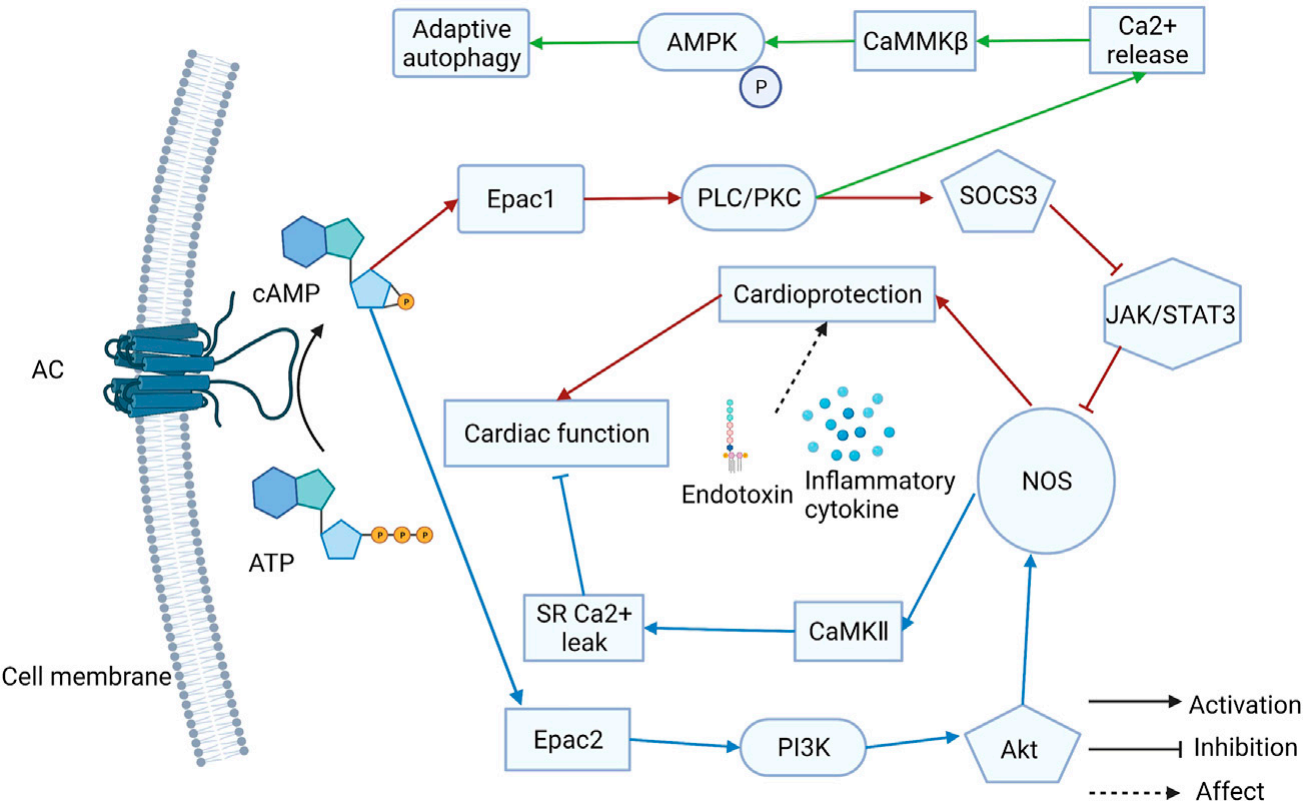
The different roles of Epac1 and Epac2 in heart failure (HF) treatment. Both Epac1 and Epac2 play different roles in arrhythmia and HF and they may become attractive targets for treatment.

Almost all cardiovascular diseases can eventually cause the onset of HF, which is the terminal stage in the development of heart disease. Fibrosis and remodeling are the typical characteristics of HF. The regulation of Ca^2+^ levels is vital for HF therapy. Anne-Coline Laurent et al. demonstrated that β-AR activates the Epac1 via cAMP, consequently activating Rap2B and PLC, which induces intracellular Ca^2+^ release. Then, because of the Ca^2+^ release, CaMMKβ is stimulated and induces AMPK phosphorylation. Ultimately, the mTOR1 is inhibited and adaptive autophagy is activated to antagonize Epac1-induced pathological cardiac remodeling (Laurent et al., 2015). Moreover, Epac1 inhibition can protect the heart from chronic catecholamine stress and pressure overload by inhibiting the phosphorylation of serine-16 and PLN (Okumura et al., 2014). Therefore, Epac1 inhibition might be a useful way to treat HF. The GRK5-CaMKⅡ axis is a potential therapeutic to combat HF (Xu et al., 2020). Additionally, a selective Epac1 inhibitor, AM-001 demonstrated pathological cardiac remodeling reduction, which is induced by β-AR activation. The AM-001 reduced the nuclear translocation of GRK5, resulting in the nuclear accumulation of HDAC5 (Laudette et al., 2019). Epac1 is an attractive target for HF induced by lipopolysaccharides. It was revealed that Epac1 overexpression in the hearts of mice can inhibit the Jak/STAT/iNOS pathway, which can restore cardiac function. Cardiac protection may manifest only when sympathetic activity is increased (Jin et al., 2017b). An earlier study found that Epac1 expression levels drop sharply after a myocardial infarction (MI) that Epac1 can reduce fibroblast collagen production (Yokoyama et al., 2008). Therefore, MI causes ventricular fibrosis, which is associated with cardiac remodeling. According to a recent study, Epac1-signaling inhibits left atrial-selective fibrosis collagen secretion and stimulating Epac1 has a strong cardioprotective effect against left atrial remodeling (Surinkaew et al., 2019). Taken together, Epac1 has a dual role in HF development. Compared to arrhythmia therapy, Epac1 plays a more complex role in HF. Although the expression of Epac2 is lower than that of Epac1 in the heart, Epac2 also influences HF development. Another study pointed out that β-AR can mediate myocyte sarcoplasmic reticulum Ca^2+^ mishandling via cAMP-Epac2-PI3K-Akt-NOS1-CaMKII resulting in heart failure and arrhythmia (Pereira et al., 2017). In summary, Epac1 and Epac2 play different roles in arrhythmia and HF development (Figure 1). These findings suggest that Epac signaling has a PKA-independent cardiac function in regulation. Both Epac1 and Epac2 may become attractive targets for HF treatment.

### Ischemia-Reperfusion Injury

**FIGURE 2:**
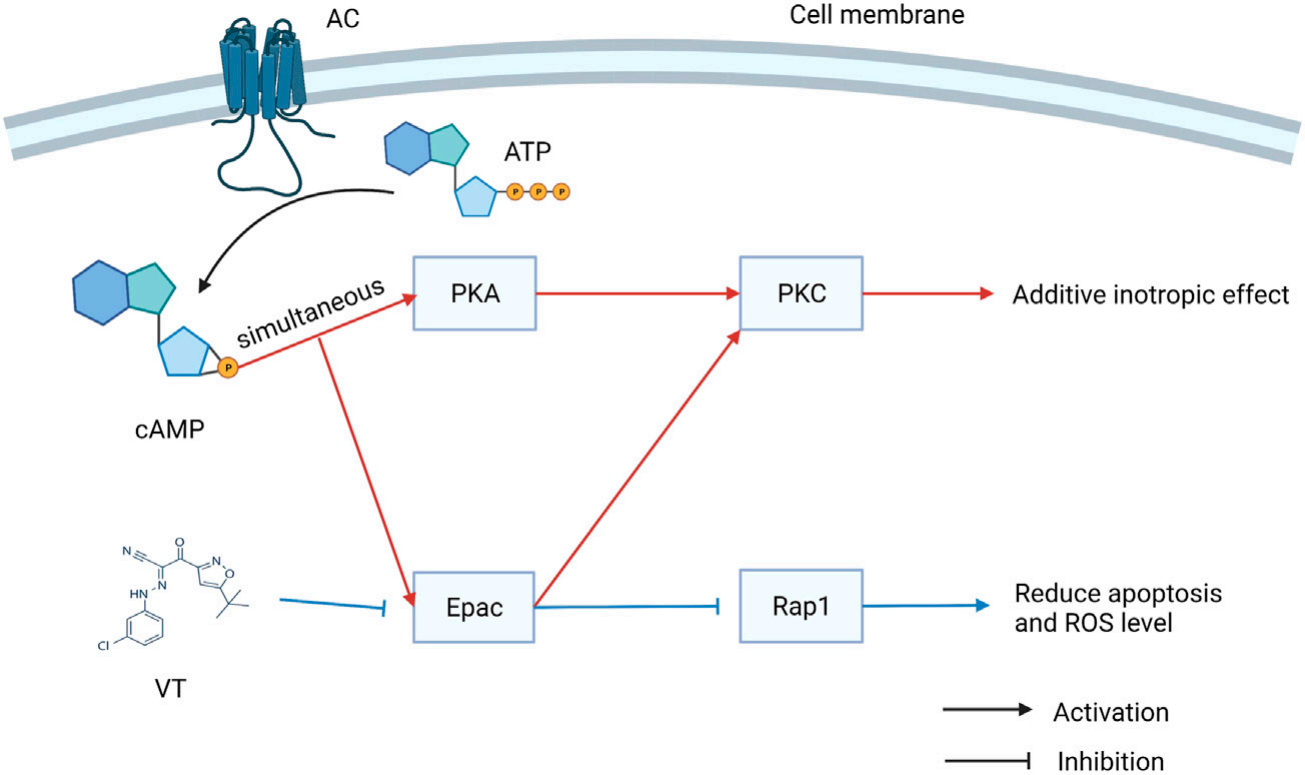
The dual role of exchange proteins directly activated by cAMP (Epac) in I/R injury. Activating Epac and PKA simultaneously could result in significant cardioprotection against I/R injury. However, VT can inhibit the Epac signal to reduce apoptosis and ROS level.

Revascularization can be used to rescue myocardial tissue after ischemic events. Reperfusion, on the other hand, may contribute to ischemia-reperfusion (I/R) injury, which is the leading cause of death worldwide, with high morbidity and mortality rates. Previous studies have mainly focused on the therapeutic potential of Epac1 inhibition. Fazal et al. found Epac1 to be regulating the opening of the mitochondrial permeability transition pore, ROS production, and Ca^2+^ uptake. I/R-induced cardiomyocyte apoptosis can be prevented by genetic ablation or pharmacological inhibition of Epac1 (Fazal et al., 2017). A recent study showed that the inhibition of Epac1 can significantly alleviate myocardial I/R injury (MIRI) by inhibiting the Epac1/Rap1/NOX4 signaling pathway (Yang H. et al., 2021). However, Khaliulin et al. observed contrasting results that activating Epac and PKA simultaneously could result in significant cardioprotection against I/R injury. PKC plays an important role in promoting cardioprotection via Epac and PKA (Khaliulin et al., 2017). Therefore, further studies are needed to corroborate the specific role of Epac1 in I/R therapy and the role of Eapc2 in I/R injury should be identified (Figure 2).

## Cerebrovascular Disease

Cerebrovascular disease is a leading cause of death and disability globally. Stroke is a common cerebrovascular disease that has a potential therapeutic target, Epac. However, Alzheimer’s disease (AD) and Parkinson’s disease (PD) can also be seen as vascular diseases and Epac also plays an important role in their therapeutic effects.

### Stroke

**FIGURE 3:**
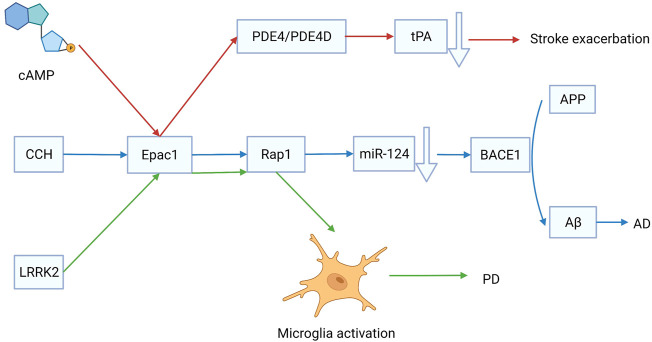
The role of exchange proteins directly activated by cAMP 1 (Epac1) in the development of stroke, Alzheimer’s disease (AD), and Parkinson’s disease (PD). Targeting Epac1 is effective for treating cerebrovascular diseases.

Stroke is a group of diseases that cause brain tissue damage due to the sudden rupture or obstruction of cerebral blood vessels. There are two main types of strokes, ischemic stroke and intracerebral hemorrhage (ICH). Ischemic stroke occurs at a higher rate than ICH. Injection of tissue plasminogen activator (tPA) is an effective treatment for ischemic stroke. An *in vitro* study showed that the expression and release of tPA are regulated by phosphodiesterase-4 (PDE4) and PDE4D, which are mediated by Epac. Therefore, targeting Epac could regulate endogenous tPA to rescue the ischemia (Yang et al., 2012) (Figure 3). Ischemic stroke can result in several serious outcomes, such as neuronal injury, retinal swelling, neuroinflammation, and blood-brain barrier (BBB) injury. According to Liu et al., after transient middle cerebral artery occlusion, Epac2^−/−^ mice showed a more severe neuronal injury than Epac1^−/−^ mice, indicating that Epac2, rather than Epac1, plays an important role in cerebral function rescue following an ischemic stroke (Liu et al., 2015). Neuronal injury is a fatal problem that requires stimulation of axonal regeneration in the remaining neurons. Epac can decrease the sensitivity of neurons to axonal grown inhibitors via induction reversible internalization of Nogo-A receptor 1 (Gopalakrishna et al., 2020). The BBB is well known to be critical in maintaining brain homeostasis. It can be disrupted after an ischemic stroke injury. The glycoprotein (GP)-Ib antagonist anfibatide can mitigate BBB injury caused by I/R injury *via* activation of the Epac/Rap1 pathway. Therefore, regulation of Epac or blockade of GP-Ib may represent therapeutic targets for ischemic stroke (Chu et al., 2021b). In conclusion, Epac activation reduced the risk of ischemic stroke.

However, in addition to ischemic stroke, Epac can also be a potential therapeutic target for ICH. ICH is associated with a high death rate and serious long-term consequences for survivors. The EP2 receptor imparts multiple beneficial effects via the cAMP/PKA signaling pathway. In contrast, the EP2 receptor can contribute to delayed neurotoxicity by enhancing chronic inflammation through the cAMP/Epac pathway. Therefore, Epac inhibition can rescue neuroinflammation for the treatment of ICH (Luo et al., 2017). However, most of the reported articles do not confirm which type of Epac is the therapeutic target, and further studies are needed to confirm this.

### Alzheimer’s Disease

AD is a common regressive disease of the central nervous system that causes many social burdens. Unfortunately, the pathogenesis of AD is ambiguous, and the disease is incurable. Chronic cerebral hypoperfusion (CCH) has been proposed as a risk factor for AD. A persistent decrease in cerebral blood flow is the main characteristic of CCH, which causes neurovascular dysfunction and cerebral ischemia/hypoxia (Popa-Wagner et al., 2015). Amyloid-β peptide (Aβ) deposition is a hallmark of AD. The β-site amyloid precursor protein cleaving enzyme 1 (BACE1) catalyzes the transformation of amyloid precursor protein (APP) to Aβ. MicroRNA, miR-124 can decrease the expression of BACE1 via activation of the Epac/Rap1 pathway (Zhang et al., 2017) (Figure 3). The expression levels of Epac1 and Epac2 decreased significantly after CCH injury. Epac may have effects on other types of vascular dementias by regulating CCH. Another hypothesis suggests that neuronal damage caused by cerebral microvasculature dysfunction could lead to AD. The cAMP/Epac signaling pathway is promising for regulating BBB permeability because it has significant implications for maintaining low endothelial permeability (Vina et al., 2021). The transcription of Epac1 elevates, while that of Epac2 decreases in the frontal cortex of patients with AD (McPhee et al., 2005). Furthermore, AD is associated with isoform-specific changes in Epac1 and Epac2 expression. In the frontal cortex of patients with AD, Epac1 elevates expression increases while Epac2 expression decreases (McPhee et al., 2005). Therefore, further studies are required to investigate the role of Epac in cerebral microvasculature dysfunction. However, early intervention on the emotionality factor can help physicians diagnose AD. Moreover, a recent study reported that Epac2 plays a more important role in mood regulation and cognitive function than Epac1 (Zhou et al., 2016). Understanding the function of Epac can thus help to identify new treatments for AD.

### Parkinson’s Disease

PD is caused by the degeneration of dopaminergic neurons located in substantia nigra pars compacta, which innervates the striatum. It is well known that PD symptoms include not only resting tremors and bradykinesia, but also cognitive deficits (Ffytche et al., 2017). L-3,4-Dihydroxyphenylalanine (l-DOPA) is normally prescribed for PD treatment. However, incorrect use of l-DOPA can accelerate the progression of PD. It is found that a toxic dose of l-DOPA can stimulate Epac-dependent sustained ERK1/2 and JNK1/2 systems, causing c-Jun phosphorylation mainly at Ser-63, and nerve cells to enter apoptosis via the caspase-3 system (Park et al., 2016). Previous studies have shown that cAMP may play an important role in the clinical treatment of PD. However, more research is needed to determine whether the decrease in cAMP and cGMP signals is inherent in PD or whether increasing cyclic nucleotide signaling can provide therapeutic relief (Kelly, 2018). Several studies have shown that leucine-rich repeat kinase 2 (LRRK-1) restricted microglial activation is involved in the initiation and progression of PD. Epac1 expression increased 11–15 folds in LRRK2^−/−^ mice compared with that in wild-type mice. It is reported that the LRRK2/Epac1/Rap1 axis can regulate the activation of innate immune functions in microglia and it is a potential therapeutic target for PD (Levy et al., 2020) (Figure 3). In conclusion, Epac promotes the progression of PD. Therefore, the inhibition of Epac may be an effective strategy for PD treatment.

## Cancer

Cancer is a leading cause of mortality and morbidity worldwide, posing a serious threat to human health. Some researchers predict that the number of patients with cancer will increase rapidly over the next 50 years. By 2070, approximately 34 million new cancer cases will be diagnosed, which is twice the number in 2018 (Soerjomataram and Bray, 2021). Currently, cancer treatment includes surgery, chemotherapy, and radiation therapy. However, due to the development of chemoresistance and relapse, these treatments are not always successful (Wehbe et al., 2020). Thus, alternative therapeutic approaches are required for cancer treatment. Epac is well-known for its dual role in the development and treatment of cancer (Kumar et al., 2018). Several studies demonstrate that Epac signaling can regulate the proliferation, migration, and metastasis of cancer cells (Wehbe et al., 2020). Moreover, the Epac signal can influence the cancer-associated angiogenesis (Kumar et al., 2018). Therefore, Epac may be an effective target for cancer treatment.

### Lung Cancer

**FIGURE 4:**
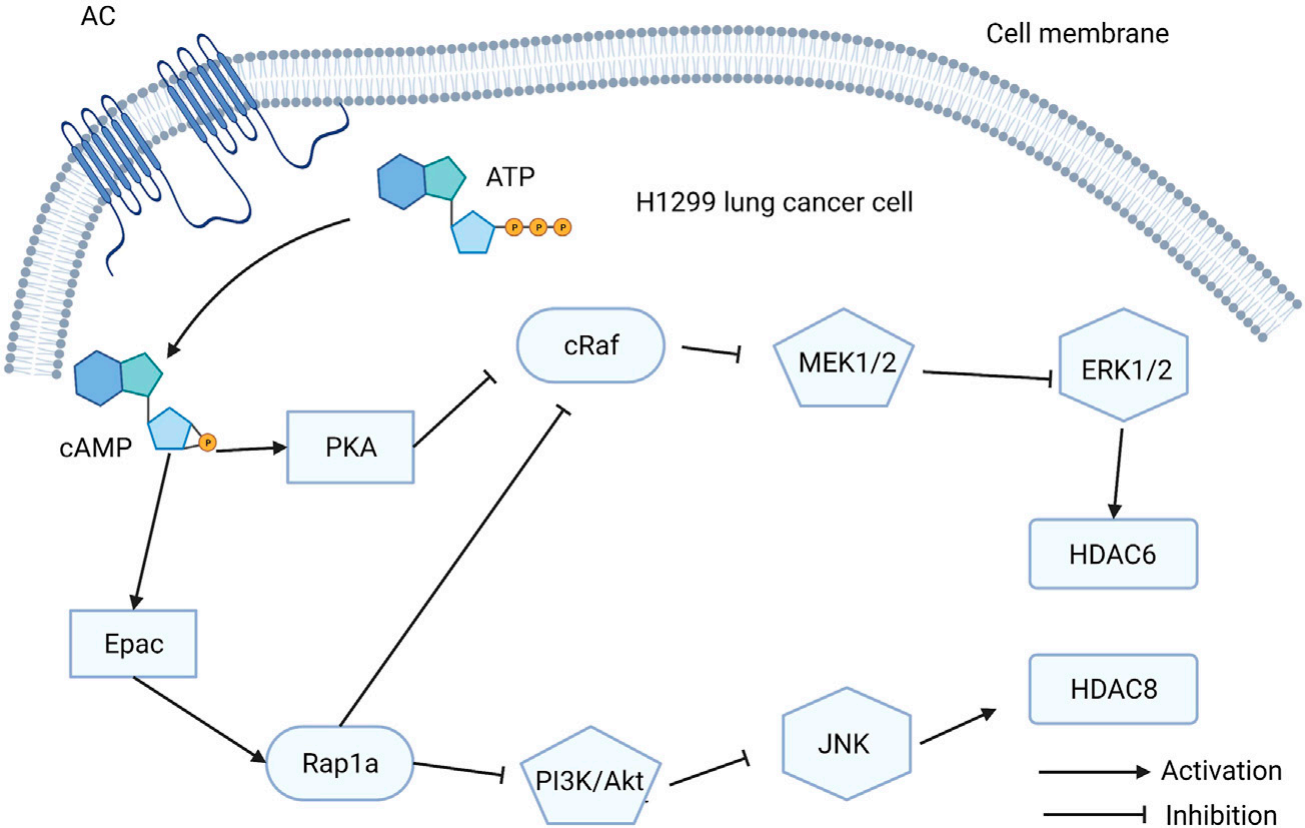
The function exchange proteins directly activated by cAMP (Epac) in H1299 lung cancer cells. Activation of Epac signaling stimulates HDAC6 and HDAC8 to promote the H1299 lung cancer cells migration.

Lung cancer is one of the most common cancers worldwide and is the leading cause of cancer-caused death (Bray et al., 2020). Histone deacetylases (HDACs) are enzymes that regulate gene transcription and cancer development by removing the acetyl groups of lysine residues in histones and non-histone proteins (Park and Juhnn, 2017). HDAC expression is regulated by cAMP. As a downstream effector of cAMP, Epac has a significant effect on HDAC expression. A recent study found that isoproterenol (ISO)-induced HDAC6 upregulation can stimulate lung cancer cell migration by inhibiting ERK via activation of PKA and Epac pathways. ISO can mimic the stress response signals that can affect pathological outcomes in various diseases (Lim and Juhnn, 2016). Another study showed that cAMP can increase HDAC8 expression in lung cancer cells by inhibiting the PI3K/Akt pathway via Epac2/Rap1A activation, which is induced by ISO (Park and Juhnn, 2017). Upregulation of HDAC6 and HDAC8 can promote cancer cell migration, which can accelerate tumor metastasis (Figure 4). However, HDAC8 promotes cisplatin-induced apoptosis in lung cancer cells by repressing Tip41 expression (Park and Juhnn, 2017).

Tumor growth is characterized by increased vascular permeability. The vascular barrier is maintained by cell-to-cell junctions. JAM-A restricts vascular permeability by increasing claudin-5 expression and Epac signaling mediates this process in the brain and lungs (Kakogiannos et al., 2020). PDE4 inhibition reduces the expression of vascular endothelial growth factor (VEGF) in lung cancer cells. Moreover, researchers demonstrated that this effect is related to the crosstalk between HIF and the PDE4/cAMP/PKA/Epac pathway (Pullamsetti et al., 2013).

In summary, Epac signaling plays a dual role in lung cancer treatment and inhibition of Epac may be a possible treatment method. Therefore, further research is needed to investigate the specific effects of Epac inhibition in lung cancer treatment.

### Pancreatic Cancer

Pancreatic cancer (PC) is a common digestive system cancer. It is associated with a high mortality rate because of its high malignancy and poor prognosis. Many surgeons refer to it as the “Cancer King” because it is difficult to diagnose and treat. Epac1 inhibition is shown to reduce the migration and invasion of PC (Almahariq et al., 2013; Almahariq et al., 2015). In addition to pharmacological inhibition, Epac1^−/−^ mice also reduced PC metastasis. Epac1 can increase the expression of integrin b1 (Itgb1), which mediates the malignant phenotype of PC (Almahariq et al., 2015). To summarize, Epac1 signaling is important in PC metastasis. Meanwhile, a recent study showed that the inhibition of MRP4 can prevent cell proliferation by inhibiting the cAMP/Epac/Rap1 pathway (Carozzo et al., 2019). Thus, the inhibition of Epac1 is a promising strategy for PC treatment.

Neovascularization is related to the aggressiveness of malignancies. However, the current anti-angiogenic strategy is ineffective in patients with PC (Li et al., 2019). Although Epac inhibition has been shown to have a strong anti-angiogenic effect (Doebele et al., 2009; Liu et al., 2020), no relevant research on the anti-angiogenic effects of Epac in PC has been conducted. Therefore, further research is needed to explore the effects of Epac on PC.

### Glioma

Glioma is a form of brain tumor with a high mortality rate. Astrocytoma is a type of glioma. Surgery is difficult because of the migratory and invasive characteristics of glioma cells and had no discernible effect on survival. Connexin 43 (Cx43), a key gap junction-forming protein, has been found to be inversely related to the degree of malignancy in glioma (Mostafavi et al., 2014; Khaksarian et al., 2015). A previous study showed that clenbuterol hydrochloride can increase Cx43 and miR-451 to suppress human astrocytoma cells via the cAMP/Epac (Mostafavi et al., 2014). Epac signaling promotes the expression of Cx43 and miR-451. However, another study reported that Epac activation can increase the levels of oncomiRs, miR-155, and miR-27a while decreasing the levels of miR-146a (Khaksarian et al., 2015). Stimulation of the Epac pathway promotes astrocytoma development. These findings indicate the dual role of Epac in cancer development and treatment.

In addition to Cx43 and miRNA, the function of this barrier is critical for glioma treatment. The blood-tumor barrier (BTB) consists of brain tumor capillaries, that can prevent anti-tumor drugs from entering the tumor tissue. The BTB hyperpermeability induced by endothelial monocyte-activating polypeptide II can be restrained by the activation of the cAMP/Epac/Rap1 signaling pathway (Li et al., 2015). Inhibition of the Epac signal plays an important role in maintaining the vascular barrier and intercellular tight junctions (Kakogiannos et al., 2020), which may have an anti-angiogenic effect in glioma. However, recent studies have focused on astrocytoma and ignored other types of gliomas, such as oligodendroglioma, ependymoma, and mixed-glioma. Thus, the therapeutic effects of Epac targeting should be investigated further.

### Prostate Cancer

Prostate cancer is an epithelial malignancy that develops in the prostate with no early symptoms. cAMP and its downstream signaling molecules, PKA and Epac, are critical for the proliferation and migration of prostate cancer cells. Epac may promote the proliferation and migration of prostate cancer cells through B-Raf/ERK and mTOR signaling cascades (Misra and Pizzo, 2009) or Ras/MAPK signaling (Misra and Pizzo, 2013). Interestingly, these effects were PKA-independent and Epac1-dependent. Epac mediates the proliferation of cancer cells, whereas PKA may have the opposite effect. Protein kinase A inhibitor proteins (PKIs) are highly expressed in prostate cancer and can redirect cAMP signaling to Epac/Rap1 and MAPK activation. Moreover, PKIs are associated with reduced survival rates (Hoy et al., 2020).

Vasoactive intestinal peptide (VIP) promotes angiogenesis, invasion, and metastasis in prostate cancer by regulating the expression of VEGF and COX-2, both of which are related to the activation of NF-κB. VIP regulates VEGF expression in prostate cancer by targeting the cAMP/Epac/ERK/PI3K signaling pathway. Moreover, these effects are PKA-independent (Fernandez-Martinez et al., 2015), demonstrating that the Epac signal can regulate angiogenesis in prostate cancer.

Epac seems to promote the development of prostate cancer. However, Epac can reduce migration and proliferation by inhibiting MAPK signaling and RhoA activation (Grandoch et al., 2009). The main reason for this was a difference in the cell culture used. In addition to the complex cell culture of prostate cancer, the lack of *in vivo* studies using animal limit these studies. Therefore, further studies with animal models are required.

### Breast Cancer

Despite recent advancements in surgical treatment for breast cancer therapy, breast cancer is still considered a complex disease. Many researchers believe that Epac1 could be a potential therapeutic target for breast cancer treatment. Enhancement of glycolysis with an increase in sugar consumption is a typical characteristic of most cancer cells. Increased sugar uptake promotes oncogenesis by activating the cAMP/Epac signaling pathway, which has been linked to the development of breast cancer (Onodera et al., 2014). Inhibiting Epac1 can reduce the ability of breast cancer cells to migrate and promote cell apoptosis, which is related to the delocalization of A-kinase anchoring protein 9 (Kumar et al., 2017). Additionally, PGE2-EP4/PKA/Epac signaling in breast tumor cells can increase mucin domain-3 (TIM-3) expression in Jurkat T cells. The increasing number of TIM-3^+^ breast cancer cases is associated with poor prognosis (Yun et al., 2019). Therefore, Epac inhibition may reduce the development of breast cancer.

Moreover, a study by Avanzato et al. showed that the activation of P2X7 and P2Y11 purinergic receptors via cAMP signaling can normalize tumor-derived endothelial cells. Instead of PKA, Epac1 may be involved in this process. This finding may help reveal the true role of purinergic agonists and Epac in tumor vessels (Munaron et al., 2017). In summary, Epac also plays a dual role in breast cancer, and more studies are needed to confirm its role in the specific type of breast cancer.

### Melanoma

Melanoma is prevalent worldwide, and the prognosis for advanced melanoma is poor with acquired resistance being a major therapeutic challenge. Baljinnyam et al. revealed the role of Epac in melanoma metastasis. Activation of Epac can regulate the production of heparan sulfate and increase syndecan-2 translocation to promote cell migration. Moreover, Epac has been shown to increase melanoma pulmonary metastasis (Baljinnyam et al., 2009). It was also reported that Ca^2+^ played a critical role in melanoma cell migration. Epac promotes actin assembly by increasing Ca^2+^ release from the endoplasmic reticulum via the PLC/IP3 receptor pathway (Baljinnyam et al., 2010). Furthermore, the expression levels of Epac1 in primary melanoma were lower than those in metastatic melanoma. Cell migration and metastasis can be reduced by ablation of Epac1. These results demonstrate that Epac1 promotes the migration of melanoma cells. The potential mechanism is associated with the upregulation of N-sulfation, which is catalyzed by N-deacetylase/N-sulfotransferase (NDST-1) (Baljinnyam et al., 2011).

Previous studies have focused on the migration of melanoma cells. Cell-to-cell communication is regulated by Epac1 via the modification of HS/FGF2 signaling. Epac1-rich melanoma cells can promote their own migration while increasing the migration of Epac1-poor melanoma cells (Baljinnyam et al., 2014). Moreover, Epac1-rich melanoma cells can promote the migration of neighboring endothelial cells, which can accelerate angiogenesis in neoplastic tissues (Baljinnyam et al., 2014). In addition to angiogenesis, the vasculogenic mimicry (VM) theory focuses on tumor development. According to Lissitzky et al., the activation of Epac/Rap1 results in VM inhibition via the production of the Rap1-GTP. Therefore, Epac has a dual effect on the nutritional access of melanoma cells (Lissitzky et al., 2009). In summary, Epac may become a therapeutic target for melanoma because of its anti-migratory effect. However, further research is needed using melanoma cells from different backgrounds.

### Other Cancers

**FIGURE 5:**
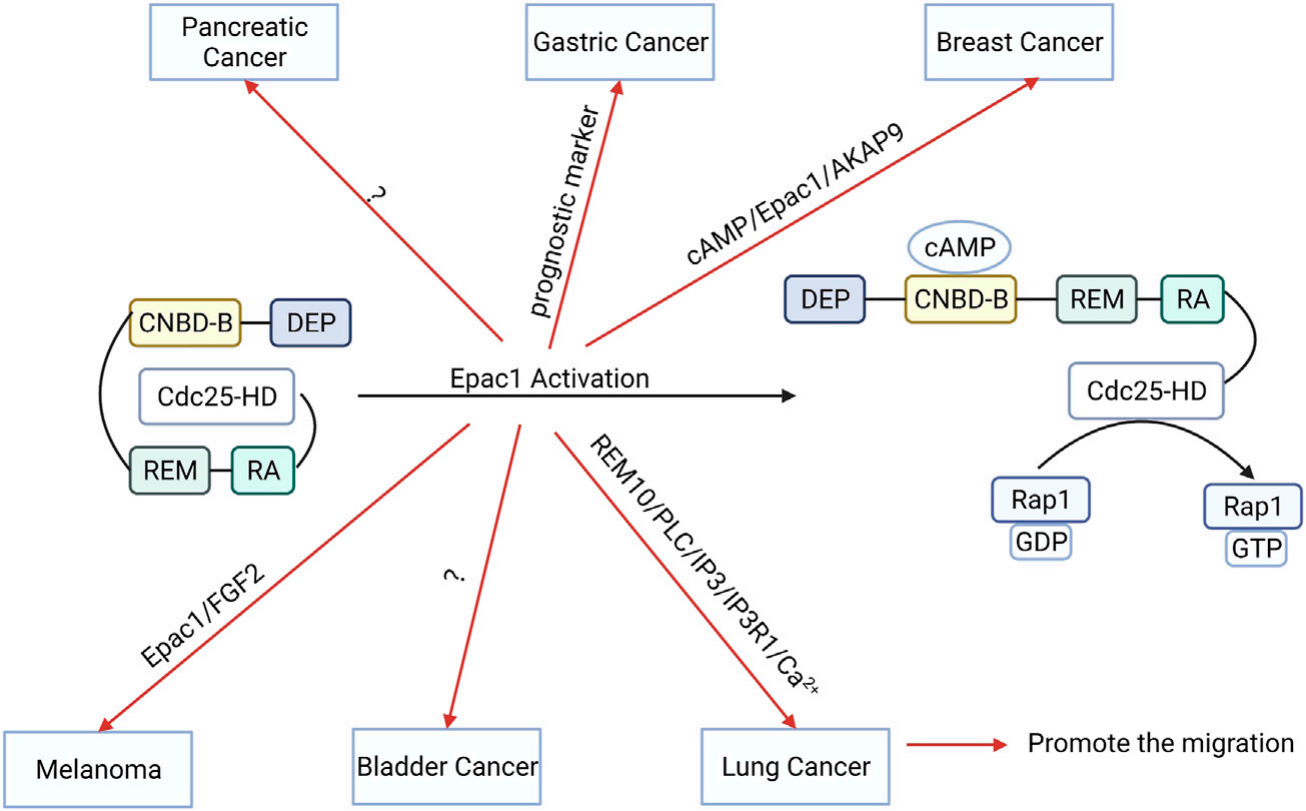
The function and potential mechanism of exchange proteins directly activated by cAMP 1 (Epac1) vary across different tumors. Activation of Epac1 can promote the migration of gastric cancer, breast cancer, bladder cancer, melanoma, lung cancer cells, and pancreatic cancer cells migration.

Epac1 is an important target in many other types of cancer as well. High expression levels of Epac1 are associated with poor prognosis for gastric cancer (GC). Epac1 targets a potential therapeutic strategy for treating GC (Sun et al., 2017). Moreover, Epac1 activation attenuated the migration of bladder cancer cells (Ichikawa et al., 2018). Epac1 expression levels are significantly increased in rectal carcinoma and Epac1 promotes the development of rectal carcinoma (Kong X. Y. et al., 2019). Therefore, these findings indicate that the function of Epac1 varies across different tumors (Figure 5). Furthermore, Epac1 regulates the migration, development, and apoptosis of cancer cells, making it a potential therapeutic target for cancer treatment. Moreover, targeting Epac may improve the treatment efficacy and prognosis because of its anti-angiogenic effect. However, the dual role of Epac signaling in cancer treatment poses a limitation for further research. Therefore, additional studies are needed to elucidate the role of Epac1 in other types of cancers.

## Kidney Diseases

Kidney diseases pose a significant risk to human health. The etiology is complex, making their diagnosis and treatment difficult. Despite their different expression levels, current studies show that both Epac1 and Epac2 are critical for kidney function adjustment (Cherezova et al., 2019; Tomilin et al., 2022). Chronic kidney disease (CKD), characterized by renal fibrosis, is a global health problem. Epac regulates cell migration, proliferation, and apoptosis by activating Rap1 (Schinner et al., 2015). Moreover, Epac plays an antifibrotic role by inhibiting TGF-β signaling (Schinner et al., 2015; Ding et al., 2018).

The activation of cAMP/Epac can improve the prognosis of CKD. Moreover, Epac is shown to regulate urine formation. Cherezova et al. reported that in Epac1^−/−^ and Epac2^−/−^ mice, the expression levels of sodium-hydrogen exchanger type 3 (NHE-3) were decreased in the proximal tubule, leading to polyuria and osmotic diuresis (Cherezova et al., 2019). Epac1-related mechanisms protect against diabetes insipidus by maintaining tight junctions in the collecting duct. Furthermore, Epac1 regulates renal papillary osmolarity to protect renal function (Asrud et al., 2020). However, another study found that inhibition of Epac can treat hypertension caused by epithelial Na^+^ channel over-activation. Although more careful and long-term studies are needed to confirm the efficacy and safety of Epac inhibitors (Tomilin et al., 2022), the potential therapeutic effect of Epac in renal diseases has attracted increasing interest from researchers.

### Ischemic Kidney Injury

All the factors that induce renal artery stenosis can cause ischemic nephropathy. Ischemic kidney disease causes irreversible damage to the renal structure, leading to renal failure. Epac signaling is found to protect renal function by activating downstream molecules, especially Rap1 signaling (Stokman et al., 2011; Stokman et al., 2014; El-Mokadem et al., 2021). Endothelial progenitor cells (EPCs) can protect the kidneys in a mouse model of acute ischemic renal failure. Patschan et al. demonstrated that the activation of Epac1 can enhance the renoprotective effects of EPCs as well as their anti-ischemic potential (Patschan et al., 2010).

Renal ischemia-reperfusion (I/R) injury is harmful to renal function and can cause acute renal failure. I/R injury is the leading cause of chronic allograft dysfunction following renal transplantation. A recent study by Geurt Stokman et al. reports that stimulating Epac/Rap can reduce ischemia-induced kidney failure. Improved tubular epithelial cell adhesion also reduces renal dysfunction (Stokman et al., 2011). Another study showed that Epac/Rap signaling reduces ROS production in the tubular epithelium while maintaining glutathione synthesis (Stokman et al., 2014). Therefore, Epac can reduce severe oxidative stress following reperfusion injury. Renal IR injury can cause structural damage and kidney dysfunction. Epac can maintain cell-cell adhesion to rescue the injured kidneys. A recent study by El-Mokadem et al. reported the therapeutic effect of the Epac-1/Rap-1 signaling pathway. Ticagrelor is an antiplatelet drug that is widely used in clinical practice that can reversibly block the purinergic receptor for adenosine diphosphate (P2Y12) (Parker and Storey, 2016). Therefore, it can enhance AC to increase cAMP. Ticagrelor can ameliorate the renal function and structure after I/R injury via the Epac1/Rap1 pathway (Boncler et al., 2019). In summary, Epac can improve renal function following an ischemic or reperfusion injury. However, research on Epac in kidney diseases is scarce, and more systematic research is needed to reveal the physiological function of Epac in the kidney.

### Renal Tumor

Renal cancer is a type of malignant tumor that originates in the urinary tubular epithelial system of the renal parenchyma. Surgical treatment is usually the preferred treatment for renal cancer. Merlin/neurofibromatosis type 2 (NF2) is a tumor suppressor, and the Merlin/NF2 mutation is the cause of the development of kidney cancer. An earlier study showed that the interactions between cAMP/Epac, cAMP/PKA, and Hippo-YAP/TAZ can regulate the metabolic network of tumor cells and promote their survival under different nutrient conditions (White et al., 2019). Cisplatin is an anti-cancer drug that causes severe nephrotoxicity due to proximal tubular epithelial cell damage. Activation of the cAMP/Epac signaling pathway protects the cell-cell junction and prevents cell apoptosis, reducing cisplatin-induced nephrotoxicity (Qin et al., 2012). However, the role of Epac in renal cancer is complex and not fully understood. Therefore, the activation or inhibition of Epac should be evaluated more carefully when designing the potential therapeutic treatments for renal tumors.

## Diabetes

Diabetes mellitus is a group of metabolic diseases characterized by hyperglycemia. Many of the available hypoglycemic drugs have decreased the mortality rate of diabetes. Regulation of insulin secretion is critical for diabetes treatment. Transmembrane receptor potential melastatin 2 (TRPM2), a potential therapeutic target for type 2 diabetes, can induce insulin secretion via cAMP/Epac signaling pathway (Yosida et al., 2014). Epac2A is abundantly expressed in the pancreas (Wehbe et al., 2020) and the Epac2A/Rap1 pathway can enhance the augmenting effect of insulin secretion by incretins and sulfonylureas (Takahashi et al., 2015). Meanwhile, patients are burdened by chronic complications of diabetes. Effective hypoglycemic therapy and blood pressure control can delay the onset and progression of diabetic microvascular lesions. However, current treatment strategies have a poor prognosis, and there is a need to investigate new targets.

Epac1 is involved in the development of diabetic microvascular lesions and can influence vascular endothelial permeability, blood-retinal barrier (BRB) permeability, and the release of inflammatory factors. Epac1 inhibition may serve as a potential therapeutic target for treating diabetic microvascular lesions.

### Diabetic Retinopathy

Diabetic retinopathy (DR) is the most common manifestation of diabetic microvasculopathy, and DR is a serious complication of diabetes. Non-proliferative diabetic retinopathy (NPDR) and proliferative diabetic retinopathy (PDR) are two types of DR that can affect a patient’s vision and even cause blindness. Clouded vision, retinal and macular edema, and vision loss are all clinical signs of NDPR, while abnormal neo-angiogenesis is a typical sign of PDR. Therefore, inhibiting the vascular growth may be a useful way to delay the onset of DR. Epac1 expression is reduced in anoxic endothelial cells and can cause endothelial dysfunction. The pharmacological activation of Epac-1 antagonizes hypoxia-induced endothelial dysfunction (Garcia-Morales et al., 2017). Increased vascular permeability is the hallmark of BRB dysfunction, which is a common pathological feature of DR. The zonula occludens-1 (ZO-1) and occludin are critical to maintaining the BRB. Epac1^−/−^ mice can reduce the expression of ZO-1 and occludin. This study also identified a new beta-adrenergic receptor agonist that increases the expression levels of endothelial cell barrier proteins by activating Epac1 (Jiang et al., 2017). Some researchers suggest that diabetic retinal ganglion injury is a pathological feature of early DR. A recent study found that the dipeptidyl peptidase IV (DPP-IV) inhibitors saxagliptin and sitagliptin can increase the mRNA and protein levels of Epac1, and alleviate neurodegeneration. These effects are related to glucagon-like peptide 1 (GLP-1) (Hernandez et al., 2017).

Typically, inflammation is a critical factor in many serious diseases. Chronic low-grade inflammation has been detected in both patients with DR and animal models, and leukocyte stasis is a critical step in early DR development. Multiple retinal glial cells directly release inflammatory cytokines and chemokines. Epac1 is shown to reduce the release of inflammatory factors, restoring normal insulin signaling and reversing retinal vascular permeability (Curtiss et al., 2018; Ramos et al., 2018). Epac1 inhibits the production of two major cytokines: IL-1 and TNFα. Downregulation of IL-1β and TNFα ameliorates insulin signaling (Curtiss et al., 2018). The activation of Epac1 may be a potential therapeutic strategy for restoring barrier properties that are destroyed by VEGF or inflammatory cytokines (Ramos et al., 2018). High-mobility group box 1 (HMGB1) is a highly conserved nuclear protein found in mammalian cells that have a strong pro-inflammatory effect. Epac1 can reduce HMGB1 through AMPK in the retinal vasculature (Jiang and Steinle, 2020). The O-GlcNAcylation of cellular proteins contributes to diabetic retinopathy development. The Epac/Rap1/OGT signaling axis can mediate angiotensin-induced O-GlcNAcylation (Dierschke et al., 2020). These findings broaden the indications for the use of traditional ACEI agents. In conclusion, Epac1 is important for DR treatment, and local Epac1 application may become a useful DR treatment strategy.

### Diabetic Nephropathy

**FIGURE 6:**
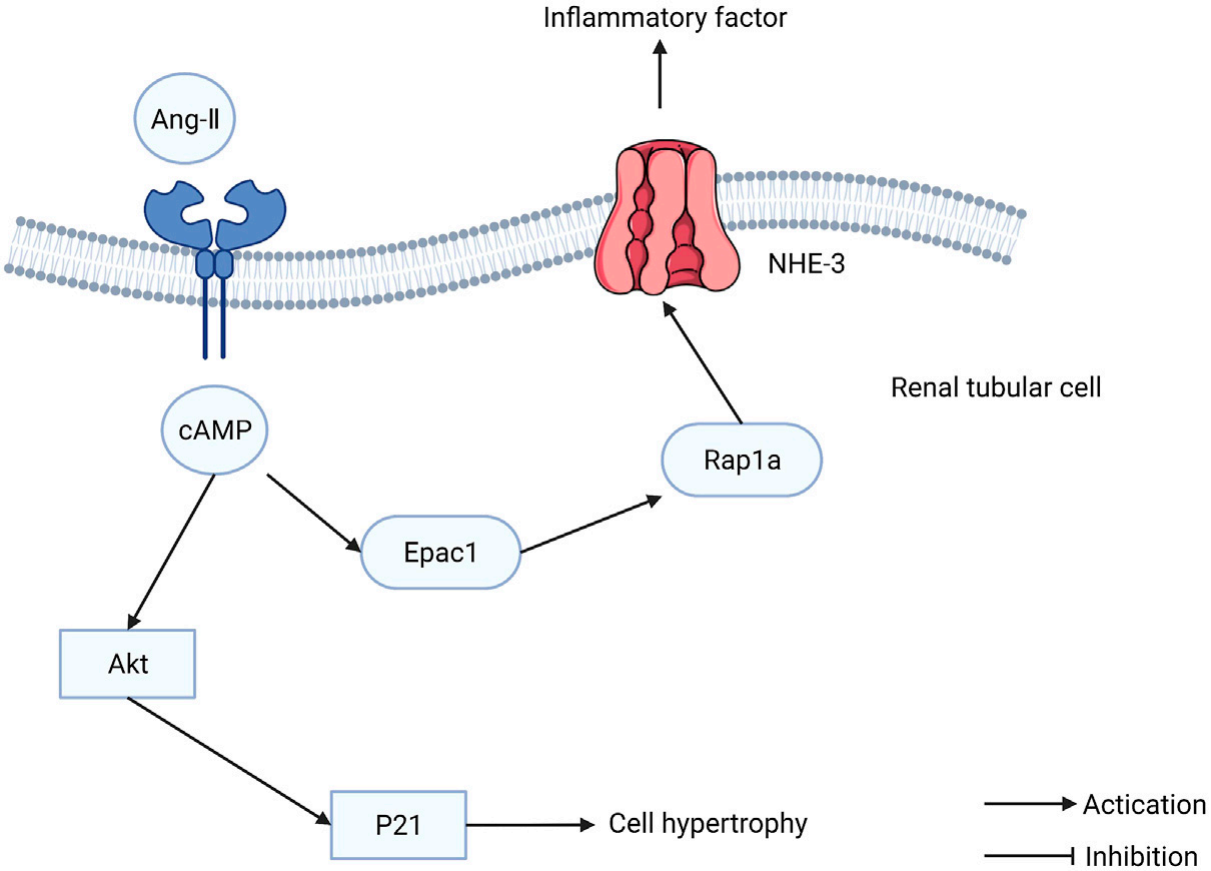
The function of exchange proteins directly activated by cAMP (Epac) in renal tubular cells. Epac1/Rap1A/NHE3 pathway can regulate the cytokines induced by Ang-II.

Diabetic nephropathy (DN) is the most severe microvascular complication of diabetes and a leading cause of end-stage renal disease. The etiology of DN is that hyperglycemia disrupts the blood vessels in the kidney, leading to dysfunction. Intensive glucose-lowering treatment can delay the development of kidney disease but cannot prevent it completely. Although blood glucose levels are maintained at a normal level, glomerular hyperfiltration and tubular hypertrophy persist. Epac1 is expressed in the kidney, particularly in cortical tubules, implying that Epac1 is involved in the pathophysiology of renal tubules. The increased transcription and translation of Epac1 increases the phosphorylation of Akt induced by high glucose, resulting in cellular hypertrophy of the renal tubules (Sun et al., 2011). The Epac1/Rap1A/NHE3 pathway can regulate the cytokines induced by angiotensin II (Ang-II) (Figure 6). Furthermore, Epac1 has been reported to influence the renin-angiotensin-aldosterone system (RAAS), which regulates the physiological function of tubular cells (Xie et al., 2014). Tubulointerstitial inflammation promotes DN development. The Epac activator can ameliorate DN tubulointerstitial inflammation via the C/EBP-β/SOCS3/STAT3 signaling pathway (Yang W. X. et al., 2021). These findings indicate that Epac1 plays a dual role in DN treatment, which is similar to the role it plays in cancer. Therefore, future studies in DN treatment should be cautious when using the Epac1 activation or inhibition strategy.

### Other Diseases

In addition to diabetic retinopathy and nephropathy, Epac also plays an important role in other serious diabetic complications. Liraglutide, a GLP-1 analog, is a commonly used anti-hyperglycemic medicine and can protect cardiomyocytes from a high-glucose environment via the Epac1/Akt signaling pathway (Wu et al., 2014). This finding confirms that Epac1 can ameliorate hyperglycemia-induced myocarditis. Painful diabetic neuropathy (PDN) is a serious complication of diabetes mellitus, and the currently used analgesic drugs pose challenges in PDN treatment. In C57BL/6J mice, Epac signaling can cause hypersensitivity to methylglyoxal (MG). PDN is ameliorated by MG scavengers and glyoxalase inducers through Epac signaling (Griggs et al., 2019). In summary, Epac plays an important role in reducing blood sugar levels and ameliorating related complications.

## Inhibitors and Activators

Inhibitors and activators of Epac were indispensable for these experiments. In previous studies, choosing the best reagent was critical, and distinguishing Epac1 and Epac2 is critical for future research. Therefore, the development of inhibitors and activators will promote an understanding of Epac signaling (Table 1).

**TABLE 1:** Inhibitors and activators of Epac.

Name	inhibitor/Activator function		References
ESI-05	Inhibitor (Epac2)	Attenuates neural apoptosis	Zhang et al. (2018); Zhuang et al. (2019)
ESI-07	Inhibitor (Epac2)	Inhibits Epac2 selectively	Tsalkova et al. (2012)
ESI-09	Inhibitor	Anti-tumor	Maeda et al. (2020)
CE3F4	Inhibitor (Epac1)	Prevents atrial and ventricular arrhythmias in mice	Prajapati et al. (2019b)
AM-001	Inhibitor (Epac1)	Protects the I/R injury	Laudette et al. (2019)
8-CPT	Activator	Alleviates inflammation and promotes endothelial cell survival	Wang et al. (2017a); Gunduz et al. (2019); Song et al. (2022)
8-CPT-AM	Activator	Attenuates VEGF signaling and restores insulin secretion	Ramos et al. (2018); Veluthakal et al. (2018)
I942	Activator (Epac1)	Reduces the vascular cell adhesion molecule 1 (VCAM1) expression in mRNA and protein levels	Wiejak et al. (2019)
SY-009	Activator (Epac1)	Activates Epac1 selectively	Beck et al. (2019)
PW0606	Activator (Epac1)	Blocks the activation of STAT3 to inhibit the IL-6 signaling to VCAM-1 induction	Wang et al. (2020)
Affimer 780A	Activator (Epac1)	Activates Epac1 selectively	Buist et al. (2021)

### Inhibitors

To date, researchers have discovered that ESI-05 and ESI-07, can inhibit the Epac2 signaling but do not activate Epac1 or PKA. The suggested mechanism is related to the difference between Epac1 and Epac2 in terms of the number and conformation of cAMP binding domains (CBD). ESI-07 can also bind to the interfaces of two CBD domains on Epac2. Moreover, ESI-07 can lock the Epac2 protein in its autoinhibitory conformation. Therefore, ESI-07 is an Epac2 selective inhibitor. As a selective Epac2 inhibitor, ESI-05 may act similarly (Tsalkova et al., 2012). In a rat model of traumatic brain injury and ICH, ESI-05 attenuated neural apoptosis (Zhang et al., 2018; Zhuang et al., 2019). Furthermore, Brefeldin A is reported to inhibit Epac2 signaling *in vivo* at concentrations up to 100 μM. However, another study by Ning Zhong and Robert S. Zucke show that Brefeldin A may have effects on other guanine exchange factors besides Epac (Zhong and Zucker, 2005). Brefeldin A does not have a direct effect on Epac and cannot inhibit Epac1 or Epac2 *in vitro* (Rehmann, 2013). Therefore, further evidence is needed to reveal the effect of Brefeldin A in the Epac1 and Epac2 exchange reactions. Additionally, ESI-09 and HJC0197, two commercially available Epac inhibitors, have protein-denaturing properties that may limit their use in experiments and ESI-09 may directly impinge on the CDC25-HD (Rehmann, 2013). However, another study proved that ESI-09 inhibits the Epac1 and Epac2 signals dose-dependently without significantly degrading proteins. Moreover, the study determined the concentrations (<20 μM) at which ESI-09 acts as an inhibitor of Epac. This study additionally demonstrated that ESI-09 is a competitive inhibitor with cAMP (Zhu et al., 2015). ESI-09 has an anti-tumor effect and is being widely used across experiments. Furthermore, ESI-09 inhibits lung cancer cells during hypoglycemia (Maeda et al., 2020) and pancreatic cancer cell growth and survival in conjunction with lithium (Wang et al., 2017b).

ESI-09 inhibits Epac1 and Epac2 simultaneously, and the lack of Epac1 selective inhibitors limits the exploration of Epac1 function. CE3F4, a tetrahydroquinoline analog, has been identified to block Epac guanine nucleotide exchange activity toward Rap1 *in vitro* (Courilleau et al., 2012). Delphine Courilleau et al. demonstrated that CE3F4 is a non-competitive inhibitor (Courilleau et al., 2013). Moreover, Rajesh Prajapati et al. found that as an Epac1 selective inhibitor, CE3F4 can prevent atrial and ventricular arrhythmias in mice (Prajapati et al., 2019b). However, the low biodisponibility limits the application of CE3F4 *in vivo*. Marion Laudette et al. identified a selective Epac1 inhibitor called AM-001. AM-001 can protect the I/R injury in acute myocardial I/R injury murine models and hypoxia-reoxygenation cardiomyocyte models. AM-001 also reduces pathological cardiac remodeling in C57BL/6 mice treated with ISO (Laudette et al., 2019). Another study showed that AM-001 can improve the contact between the CDC25-HD and CNBD domains which stabilizes the inactive conformation (Bufano et al., 2020; Yang et al., 2021a; Zhang et al., 2021).

### Activators

8-(4-Chlorophenylthio)-2′-O-methyladenosine-3′, 5′-cyclic monophosphate (8-pCPT-2′-O-Me-cAMP, 8CPT) was used to discriminate between the roles of Epac and PKA. 8CPT can activate both Epac1 and Epac2, but it tends to activate Epac1 more than Epac2 (Luchowska-Stanska et al., 2019). 8CPT was reported to alleviate inflammation in an acute murine lung injury model (Wang et al., 2017a) and an Il-10^−/−^ Crohn’s disease murine model (Song et al., 2022). In addition, 8CPT also promotes endothelial cell survival (Gunduz et al., 2019).

The membrane permeability of 8CPT is poor, and it can be improved by 8-pCPT-2′-O-Me-cAMP-AM (8CPT-AM) (Luchowska-Stanska et al., 2019). 8CPT-AM attenuates VEGF signaling and delays the development of diabetic retinopathy (Ramos et al., 2018). Moreover, 8CPT-AM can restore insulin secretion (Veluthakal et al., 2018). In summary, 8CPT and 8CPT-AM are the two main activators of Epac. Moreover, in addition to 8CPT and 8CPT-AM, ticagrelor also can activate the Epac1/Rap1 signaling pathway (El-Mokadem et al., 2021).

Recently, several selective small-molecule Epac activators have been identified. The first non-cyclic-nucleotide small molecule Epac1 selective agonist, I942, was identified using high throughput screening (HTS), nuclear magnetic resonance (NMR), and guanine nucleotide exchange factor (GEF) assays (Parnell et al., 2017). Hongzhao Shao et al. found that I942 can interact with Epac1 in the phosphate-binding cassette (PBC) and base binding region (BBR). However, the limitation of the I942 agonism is the disengagement of the CNBD hinge helix (Shao et al., 2020). Therefore, future work could improve the potency and efficacy of Epac1 activator in the foundation of the I942 structure. Furthermore, I942 can reduce the vascular cell adhesion molecule 1 (VCAM1) expression in mRNA and protein levels in human umbilical vascular endothelial cells (HUVECs) (Wiejak et al., 2019). A selective Epac1 activator, SY-009, was identified, which has a different chemical structure than I942. However, further research is warranted to determine whether SY-009 can bind to the CNBD of CNG or other cellular CNBDs (Beck et al., 2019). A series of small-molecule Epac1 activators have been compiled with the compound 25u (PW0606) being the most potent selective Epac1 binder in this series, more active than I942. Moreover, PW0606 can block the activation of STAT3 to inhibit the IL-6 signaling to VCAM-1 induction (Wang et al., 2020). Another study by Hanna K. Buist et al. identified five selective Epac1 Affimer binders, of which the Affimer 780A has a potential binding site in the CNBD of Epac1, and Affimer 780A can potentially be used in future studies of Epac1 (Buist et al., 2021).

## Conclusion and Future Perspectives

In recent years, the understanding of Epac signaling has grown rapidly. Epac has many extensive and important effects in most tissues and cells and cooperates with other cAMP effectors to regulate intracellular cAMP function. Epac and PKA are two major cAMP effectors that act synergistically, antagonistically, or independently. It is evident that Epac mediates many PKA-independent functions.

The development of pharmacological tools and animal genetic models has provided a better understanding of the functions of Epac1 and Epac2. The Epac protein is not required for normal development and survival since there are no significant physiological abnormalities in Epac1^−/−^, Epac2^−/−^, and Epac^−/−^ mice. These findings suggest that the targeted toxicity of inhibition by Epac is minimal (Robichaux and Cheng, 2018). These findings provide exciting opportunities for the development of novel therapeutic therapies. From this review, we can conclude that Epac is a potential therapeutic target for vascular diseases and that targeting Epac may be a useful therapeutic strategy for intractable diseases.

However, few gaps in literature remain. Firstly, most studies have focused on cardiovascular disease and cancer. Subsequent drug development is challenging in these two disease categories where Epac plays a dual role. Secondly, little is known about the transcription, translation, and post-translational regulation of Epac. Human genetic information and relevant clinical data on Epac proteins are still lacking, impeding the understanding of Epac function. Thirdly, it remains a challenge for researchers to discriminate the physiological function of Epac isoforms since many studies continue to use the Epac-selective cAMP analogs. Nonetheless, the development of CRISPR/Cas9, conditional Epac^−/−^ animal models, and other pharmacological tools have enabled researchers to define the physiological function of Epac1 and Epac2 on a tissue, cellular and molecular level. Finally, Epac is a promising target with high clinical value for the development of treatment strategies for vascular diseases.

## Glossary

8 CPT: 8-(4-Chlorophenylthio)-2'-O-methyladenosine-3', 5'-cyclic monophosphate
8 CPT-AM: 8-pCPT-2'-O-Me-cAMP-AM
AC: adenylyl cyclase
ACEI: angiotensin-converting enzyme inhibitors
AD: alzheimer's disease
AF: atrial fibrillation
Akt: protein kinase B
AMPK: adenosine 5‘monophosphate-activated protein kinase
Ang-II: angiotensin-II
AP: action potential
APP: amyloid precursor protein
ATP: adenosine triphosphate
Aβ: Amyloid-β peptide
BACE1: β-site amyloid precursor protein cleaving enzyme 1
BBB: blood-brain barrier
BBR: base binding region
BRB: blood-retinal barrier
BTB: blood-tumor barrier
CaMKII: Ca2+-Calmodulin-dependent protein kinase
cAMP: cyclic adenosine monophosphate
CBD: cAMP binding domains, CBD cAMP binding domains
CCH: chronic cerebral hypoperfusion, CCH chronic cerebral hypoperfusion
Cdc25-HD: cell division cycle 25 homology domains, Cdc25-HD cell division cycle 25 homology domains
cGMP: 3′5′-guanosine monophosphate
CKD: chronic kidney disease
CNB: cyclic nucleotide-binding domain
CNBD: cAMP-nucleotide binding domain
CNG: cyclic nucleotide-gated ion channels
COX-2: cyclooxygenase-2, COX-2 cyclooxygenase-2
Cx43: connexin 43, Cx43 connexin 43
DN: diabetic nephropathy
DPPIV: dipeptidyl peptidase IV
DR: diabetic retinopathy
EBP-β: enhancer-binding protein β
EP2: prostaglandin E2
EP4: prostaglandin E4
Epac: exchange proteins directly activated by cAMP
EPCs: endothelial progenitor cells
ERK1/2: extracellular-regulated kinase
ESI: Epac selective inhibitor
FGF2: fibroblast growth factor
GC: gastric cancer
GDP: guanosine-5'-diphosphate
GEF: guanine nucleotide exchange factor
GLP-1: glucagon-like peptide 1
GP: glycoprotein
GTP: guanosine triphosphate
HDACs: histone deacetylases
HF: heart failure
HMGB1: high-mobility group
HS: heparan sulfate
HTS: high throughput screening
HUVECs: human umbilical vascular endothelial cells
I/R: ischemia-reperfusion
ICH: intracerebral hemorrhage
IL: interleukin
iNOS: inducible nitric oxide synthase
IP3: inositol triphosphate
ISO: isoproterenol
Itgb1: integrin b1
Jak: janus kinase
JAM-A: junctional adhesion molecule-A, JAM-A junctional adhesion molecule-A
JNK1/2: c-Jun N-terminal kinase, JNK1/2 c-Jun N-terminal kinase
L-DOPA: L-3,4-dihydroxyphenylalanine
LOX1: low-density lipoprotein receptor 1, LOX1 low-density lipoprotein receptor 1
LRRK2: leucine-rich repeat kinase 2, LRRK2 leucine-rich repeat kinase 2
LTCC: L-type calcium channels
MAPK: mitogen-activated protein kinase
MG: methylglyoxal
MI: myocardial infarction
miR: microRNA
MIRI: myocardial I/R injury
mTOR: mammalian target of rapamycin
NDST1: N-deacetylase/N-sulfotransferase
NF2: neurofibromatosis type 2
NF-κB: nuclear factor κB
NHE-3: sodium-hydrogen exchanger type 3
NMR: nuclear magnetic resonance
NOS1: nitric oxide synthase 1
NOX4: NADPH oxidase 4
NPDR: non-proliferative diabetic retinopathy
OGT: O-GlcNAc transferase
PBC: phosphate binding cassette
PC: pancreatic cancer
PD: parkinson's disease
PDE2: phosphodiesterases2
PDE4: phosphodiesterase-4
PDN: painful diabetic neuropathy
PDR: proliferative diabetic retinopathy
PI3K: phosphatidylinositol 3-kinase
PKA: protein kinase A
PKC: protein kinase C
PKI: protein kinase A inhibitor proteins
PLC: phospholipase C
RAAS: renin-angiotensin-aldosterone system
Ras: renin-angiotensin system
REM: Ras-exchange motif
RhoA: rhotekin
ROS: reactive oxygen species
SMC: smooth muscle cell
SOCS3: suppressor of cytokine signaling-3
SR: sarcoplasmic reticulum
STAT: signal transducers and activators of transcription
TIM-3: mucin domain-3
TNF: tumor necrosis factor
tPA: tissue plasminogen activator
TRPM2: transmembrane receptor potential melastatin 2
VCAM1: vascular cell adhesion molecule 1
VEGF: vascular endothelial growth factor
VIP: vasoactive intestinal peptide
VM: vasculogenic mimicry
VT: vitexin
ZO-1: zonula occluden 1,
β-AR: β-adrenergic receptor
